# Risk Factors and Prediction Models for Venous Thromboembolism in Ambulatory Patients with Lung Cancer

**DOI:** 10.3390/healthcare9060778

**Published:** 2021-06-21

**Authors:** Ann-Rong Yan, Indira Samarawickrema, Mark Naunton, Gregory M. Peterson, Desmond Yip, Salvatore De Rosa, Reza Mortazavi

**Affiliations:** 1 School of Health Sciences, Faculty of Health, University of Canberra, Canberra 2617, Australia; ann-rong.yan@canberra.edu.au (A.-R.Y.); mark.naunton@canberra.edu.au (M.N.); g.peterson@utas.edu.au (G.M.P.); desmond.yip@anu.edu.au (D.Y.); 2 School of Nursing, Midwifery and Public Health, Faculty of Health, University of Canberra, Canberra 2617, Australia; Indira.Samarawickrema@canberra.edu.au; 3 College of Health and Medicine, University of Tasmania, Hobart 7005, Australia; 4 Department of Medical Oncology, The Canberra Hospital, Garran 2605, Australia; 5 ANU Medical School, Australian National University, Canberra 0200, Australia; 6 Department of Medical and Surgical Science, Magna Graecia University, 88100 Catanzaro, Italy; saderosa@unicz.it; 7 Prehab Activity Cancer Exercise Survivorship Research Group, Faculty of Health, University of Canberra, Canberra 2617, Australia

**Keywords:** venous thromboembolism, lung cancer, thromboprophylaxis, anticoagulant, risk prediction, risk assessment, risk model, prediction rules, risk factors

## Abstract

Venous thromboembolism (VTE) is a significant cause of mortality in patients with lung cancer. Despite the availability of a wide range of anticoagulants to help prevent thrombosis, thromboprophylaxis in ambulatory patients is a challenge due to its associated risk of haemorrhage. As a result, anticoagulation is only recommended in patients with a relatively high risk of VTE. Efforts have been made to develop predictive models for VTE risk assessment in cancer patients, but the availability of a reliable predictive model for ambulate patients with lung cancer is unclear. We have analysed the latest information on this topic, with a focus on the lung cancer-related risk factors for VTE, and risk prediction models developed and validated in this group of patients. The existing risk models, such as the Khorana score, the PROTECHT score and the CONKO score, have shown poor performance in external validations, failing to identify many high-risk individuals. Some of the newly developed and updated models may be promising, but their further validation is needed.

## 1. Background and Introduction

Cancer is a major risk factor for venous thromboembolism (VTE), which includes deep vein thrombosis and pulmonary embolism. VTE has an annual incidence of around 0.5% in cancer patients compared to around 0.1% in the general population [1]. The incidence of VTE in patients with cancer varies with cancer type, stage, and aggressiveness [2]. Among all cancer types, lung cancer has the second highest risk of VTE [3]. In cohort studies, the incidence of VTE in patients with lung cancer receiving chemotherapy was variously reported as 16.8% at three months and 14.1% at six months after the start of chemotherapy [4], and 13.9% after a median follow-up period of 12 months [5]. Having a VTE is a significant predictor of death within 2 years in patients with primary lung cancer, with hazard ratios (HRs) of 2.3 (95% CI 2.2–2.4) and 1.5 (95% CI 1.3–1.7) for non-small cell lung cancer (NSCLC) and small cell lung cancer (SCLC), respectively [6]. This matter highlights the importance of the identification of patients at risk of developing VTE so that therapeutic or preventive measures are implemented in a timely manner.

Thromboprophylaxis is suggested in hospitalised patients with lung cancer and those undergoing surgery, but the use of primary prevention of VTE in ambulatory patients with lung cancer is still debatable [7]. Choice of the anticoagulation therapy is particularly challenging in patients undergoing antineoplastic chemotherapy. On one hand, these patients are at risk of VTE over the course of therapy and beyond. On the other hand, anticoagulation is associated with a high bleeding risk, which could be life-threatening [8]. Low-molecular-weight heparins (LMWH) can reduce the risk of VTE, but current practice guidelines do not recommend their routine use, while direct-acting oral anticoagulants (DOACs) are an interesting alternative to LMWH in cancer patients [9,10,11]. Recent studies have confirmed their efficacy and safety in these patients [12]. Scientific societies such as the National Comprehensive Cancer Network (NCCN), the International Society on Thrombosis and Haemostasis (ISTH), and more recently, the American Society of Clinical Oncology (ASCO) and The International Initiative on Thrombosis and Cancer (ITAC) have supported the use of DOACs [9,10,11]. Nevertheless, the use of DOACs in this scenario should be carefully weighed against the bleeding risk, as evidence for higher risks of bleeding has emerged in studies of the general cancer population [13] and in patients starting chemotherapy [14]. As a result, it is recommended that anticoagulation is only offered to patients with a high risk of VTE, and for this we need to have robust and reliable risk assessment tools [15]. This requires a thorough understanding of the VTE risk factors and clinical prediction models to identify high-risk patients.

Clinical prediction models are epidemiological/statistical tools, which use a small number of parameters (related to the individual, or the disease, or the treatment) to estimate a likelihood in which a specific outcome (e.g., VTE) could happen. Prediction models can help clinicians better understand the individual patient’s conditions or risks, so they are able to devise a personalised treatment regimen for them [16]. In 2008, Khorana et al. established a predictive model to assess individual risk of VTE in ambulatory cancer patients receiving chemotherapy [11]. With this model, patients are assigned to one of three risk groups: low (score = 0), intermediate (score = 1–2) and high risk (score ≥ 3) [17]. Using this model, patients with lung cancer are stratified as either intermediate or high risk of developing VTE [17]. The pooled data from 45 studies including various types of cancer showed that only 23.4% (95% CI: 18.4–29.4%) of the patients who developed VTE in the first six months had been classified as being at high risk according to the Khorana score [18]. This poor performance led to the development of several modifications for the Khorana score over the years, including the Vienna Modification [19], PROTECHT [20], CONKO [21], and COPASS-CAT [22], with varying degrees of predictive ability. In this article, we have reviewed the risk factors for VTE in ambulatory patients with lung cancer, discussed some main risk assessment models for VTE in this group of patients, and reflected upon advantages and disadvantages of the models. We have also explored literature gaps and provided suggestions for further research.

## 2. VTE Risk Factors in Ambulatory Patients with Lung Cancer

Risk factors for VTE are grouped into three categories: patient-related, cancer-related, and biomarkers [1]. Regarding the patient-related risk factors for VTE, co-morbidities such as atrial fibrillation, chronic kidney disease [23], cardiovascular conditions, and overweight or obesity [24] increase the risk of VTE. Smoking [25] and recent hospitalisation [23,24] also raise the risk of VTE. It is believed that the Asian race has a lower risk than other races [26]. Factor V Leiden and prothrombin 20210A mutations are relatively common in Caucasians whereas they are very rare in Asians [27,28]. These mutations have been identified as additional risk factors for VTE in cancer patients [3]. Cancer patients carrying Factor V Leiden mutation or prothrombin 20210A mutation had a 12.1-fold (95% CI 1.6–88.1) and2.3-fold (95% CI 1.6–3.3) higher risk of VTE, respectively [3].

Similar to variations in the observed VTE risk among other types of cancer [3], some subtypes of lung cancer have higher risks of VTE compared to others; for example, lung adenocarcinoma had a higher risk of VTE occurrence than squamous cell carcinoma [6]. However, within the NSCLC group, the association of oncogene mutations with the risk of VTE is debatable. A systematic review by Liu et al. of 20 retrospective studies showed that anaplastic lymphoma kinase (ALK) mutation has a higher risk of VTE than epidermal growth factor receptor (EGFR) mutation [29], while another systematic review by Alexander et al. reported that EGFR mutation was the strongest risk factor for VTE in patients with lung cancer [30]. Several studies showed patients with an ALK mutation had a higher VTE risk than those without [31], with a hazard ratio of 2.47 (95% CI 1.04–5.90) for VTE in a median follow-up period of 7.5 months (95% CI 3.1–15.4 months) [32] or increasing the risk of VTE by 3 to 5 times over a median follow-up period of 22 months comparing to the general NSCLC population [33]. One possible reason is the expression of tissue factor (TF) gene was elevated in around 41.7% of ALK-positive, but only 11.5% of ALK-negative tissue in patients with lung cancer (*p* = 0.015) [34]. On the other hand, the prospective interventional studies using ALK inhibitor found a lower incidence of VTE than that in retrospective studies, which may be due to reduced use of chemotherapy [35].

Cancer treatment is a strong risk factor for VTE, and commonly used chemotherapy drugs can increase the risk of this clinical condition [25,26,36]. For example, gemcitabine-based chemotherapy increases the risk of VTE, with a reported odds ratio of 3.37 (95% CI 1.09–10.39) [23]. In addition, VTE was more likely to occur within the first six months of the commencement of standard chemotherapy [24]. In terms of the mechanisms involved, they are probably related to vascular endothelial damage caused by chemotherapy, especially in patients who receive the medications through central venous catheterisation over a long period [25,26,36].

There are a limited number of studies which have investigated the impacts of novel anti-cancer treatments on the haemostatic system. For instance, in patients treated with immune checkpoint inhibitors (ICIs), the cumulative incidence of VTE over a median follow-up of 8.5 months was 12.9% (95% CI 8.2–18.5%) [37]. Sato et al. found that in patients with NSCLC, anti-programmed cell death 1 (PD-1)/programmed cell death ligand 1 (PD-L1) monoclonal antibodies might impose procoagulant effects on the haemostatic system [38]. It has been reported that the activation of T-cells in vitro induced the production of TF in high-PD-L1-expressed monocytes [38]. Additionally, in cancer patients receiving ICIs, some biomarkers such as vascular cell adhesion molecule 1 (sVCAM-1) and interleukin 8 (IL-8) were associated with the occurrence of VTE [39]. The administration of ICIs and associated biomarkers have not been investigated in a VTE risk model yet, and they may be considered as candidates for a new predictive model, although the feasibility of the measurements could be a problem in clinical settings.

Some tissue or blood biomarkers have been found to have significant associations with hypercoagulable conditions in patients with cancer; therefore, they could be considered as candidate predictors in VTE risk assessment. For instance, platelets and von-Willebrand factor (vWF) not only play vital roles in preventing blood loss following a blood vessel injury by facilitating coagulation [40], but also contribute to thrombosis as well as cancer metastasis [15,41]. It has been reported that high platelet counts and/or increased platelet activity can increase the risk of developing VTE in patients with cancer [17,42]. Additionally, elevated levels of plasma vWF, particularly the high molecular weight multimers, can induce the formation of vWF-mediated platelet thrombi by facilitating platelet aggregation under high shear rate conditions [43]. In different studies, high plasma vWF was associated with the occurrence of VTE in cancer patients [44,45]. Meanwhile, increased levels of thrombin-antithrombin III complex (TAT) (mean 4.7 [95% CI 4–6] vs. 2.5 [95% CI 2–3] μg/L, *p* < 0.0001) and elevated prothrombin fragment 1+2 (F1+2), as a by-product of thrombin generation (mean 267 [95% CI 244–289] vs. 183 [95% CI 171–196] pmol/L, *p* < 0.00001) have been reported in primary lung cancer patients with VTE [46]. In addition, increased levels of D-dimer and plasmin-α_2_-antiplasmin inhibitor complex (PIC) [47], generated in fibrinolysis, and serum ionised calcium have shown associations with the occurrence of VTE in patients with lung cancer [48]. Other biomarkers or laboratory parameters, such as fibrinogen, activated partial thromboplastin time (aPTT) and albumin, showed associations with VTE [49,50], but are lacking external validation of their predictive roles. Most biomarkers, as predictors of VTE, have been typically used at the diagnosis of cancer or before the beginning of chemotherapy, whereas D-dimer has also been investigated in longitudinal studies following the diagnosis [30,51]. Some biomarkers have been studied as predictors of VTE and have been used for prediction model development. For example, platelet count, white cell count and haemoglobin have been incorporated into the Khorana score [17], although their effects on the occurrence of VTE in patients with lung cancer are controversial [30].

Inflammatory mediators (particularly those cytokines which are associated with vascular inflammation) are another group of biomarkers which could be used as predictors for new VTE prediction model development. Cancer cells produce and secrete inflammatory cytokines such as interleukin 1 (IL1), tumour necrosis factor alpha (TNF-α) and vascular endothelial growth factor (VEGF), which mediate interactions between cancer cells and host cells, including endothelial cells, platelets, monocytes and neutrophils [52]. Furthermore, the blood levels of intracellular granular proteins or surface-expressed proteins released from the activated host cells [15], including procoagulant platelets [53], may be used as predictors of a hypercoagulable status in patients with solid tumours [52]. For instance, activated monocytes in cancer patients produced more TF than resting monocytes [15]. Additionally, neutrophil elastase (NE) released by activated neutrophils showed correlations with aPTT, D-dimer, TAT, PIC and fibrinogen levels in lung cancer patients, with stronger correlations seen in NSCLC [54].

## 3. Risk Prediction Models for VTE in Patients with Cancer

Risk prediction models, also referred to as risk assessment tools or clinical prediction rules, are prognostic models, which use predictors to estimate the probability for individuals to develop a condition in the future [55]. This review covers the particulars of some available VTE risk prediction models which have been developed and/or validated in ambulatory patients with lung cancer. A summary of the main features can be found in Table 1.

### 3.1. The Khorana Score

The Khorana Score is the first and most frequently investigated VTE risk prediction model in patients with cancer. It uses the following five predictors: cancer site, platelet count, leucocyte count, haemoglobin, and body mass index (BMI). In assessing an individual’s VTE risk, 2 points are assigned for very high-risk cancer types (e.g., stomach and pancreas), 1 point for high-risk cancer (e.g., lung, lymphoma, gynaecological, bladder, testicular), 1 point for baseline platelet count ≥ 350 × 10^9^/L, 1 point for baseline leukocyte count > 11 × 10^9^/L, 1 point for baseline haemoglobin level < 100 g/L or the use of erythropoietin, and 1 point for BMI ≥ 35 kg/m^2^ (Table 1) [17]. The main advantage of the Khorana score is that all the predictors used are among the routinely measured clinical or laboratory parameters. Additionally, this score has a high specificity. However, the score has a poor sensitivity. Multiple studies showed a low sensitivity of 10–25%, a high specificity of 76–100%, and a poor discrimination (C-index) of about 0.50 with the high-risk threshold of 3 points in ambulatory patients with lung cancer [23,25,51,56,57].

On the other hand, a subgroup analysis between lung cancer and non-lung cancer patients (including colorectal, pancreatic, stomach, ovarian, breast, brain and bladder cancers) showed a different discriminatory performance for the Khorana score [58]. The dichotomous Khorana score did not identify higher risk of VTE compared with low–moderate risk in ambulatory patients with lung cancer in a meta-analysis (OR = 1.1, 95% CI 0.72–1.7), whereas it was a strong predictor for other types of cancers (OR = 3.2, 95% CI 1.8–5.6, *p* for interaction = 0.002) [58]. This finding emphasises the need for a better VTE model for ambulatory patients with lung cancer.

Recently, a change in the high-risk threshold from 3 to 2 of the Khorana score was introduced and used in randomised controlled trials of direct inhibitors of Factor Xa in ambulatory patients with cancer at a high risk of VTE [9,61]. In the stratified group with a Khorana score ≥ 2, apixaban lowered incidence of VTE with increasing major bleeding risk [9], while rivaroxaban did not lower incidence of VTE significantly [61]. This cut-off value of the Khorana score has been adopted in the latest ASCO guidelines for thromboprophylaxis in cancer (recommended for patients with a Khorana score of 2 or higher) [10]. However, so far, the latter threshold is yet to be validated in a homogeneous lung cancer population.

### 3.2. Modifications of the Khorana Score

Since its introduction in 2008, the Khorana model has been modified multiple times by the addition and/or replacement of biomarkers. For example, by adding D-dimer and soluble P-selection to it, the Vienna Modification (CATS score) was introduced in 2010 [19]. Later, by the addition of treatment-related factors, such as gemcitabine or platinum-based chemotherapy, the PROTECHT score was developed [20]. By the addition of World Health Organisation (WHO) performance status and omission of BMI, the CONKO score was developed in patients with advanced pancreatic cancer [21]. More precisely, the PROTECHT score adds 1 point for each of gemcitabine or platinum chemotherapies, and the CONKO score removes BMI but adds the Eastern Cooperative Oncology Group (ECOG) performance status (PS) ≥ 2 for 1 point, while the cut-off value is still 3 points in both scores (Table 1).

The PROTECHT score and the CONKO score have been validated in ambulatory patients with lung cancer [23,51]. Compared to the Khorana score, more patients (22–48%) were categorised into the high-risk group by the CONKO score and even more so by the PROTECHT score (52–64%), but there was no significant difference in the incidence of VTE between high- and low-risk groups stratified by either of these models. A C-index around 0.50 consistently showed a poor discrimination for both models [23,51].

The VTE risk prediction model developed by Ferroni et al. is another extension of the Khorana model with an additional predictor, which is high-sensitive D-dimer (Table 1) [59]. According to the Khorana score, patients with lung cancer are given a score of 1 point or more, having either intermediate or high risks for VTE [17]. This model is not applicable to all ambulatory patients with lung cancer, but only to those with an intermediate risk of VTE stratified by the Khorana score. A high-sensitive (HS) D-dimer level is used to select patients with a higher risk of VTE from those within the intermediate risk group stratified by a Khorana score of 1–2 points [59]. This study compared the relatively high-risk subgroup to the relatively low risk subgroup within the Khorana intermediate risk group. With a threshold of 1500 ng/mL for HS D-dimer at baseline, both sensitivity and specificity were moderate at 81.3% and 68.5%, respectively, with a moderate discriminating capacity of 0.704 [59].

There are also some other types of VTE risk models, such as a simple model with only two factors including distant metastases and platinum therapy [62], and a model using cancer site and continuous D-dimer concentration rather than a cut-off value, called the CATS-MICA model [63]. Neither of these risk prediction models has been externally validated in patients with lung cancer.

### 3.3. The COMPASS-CAT Score

The COMPASS-CAT score is a more complicated model with both cancer-related and patient-related factors, which are anthracycline treatment (6 points), time since cancer diagnosis ≤ 6 months (4 points), central venous catheter use (3 points), advanced stage of cancer (2 points), cardiovascular risk factors present (5 points), recent hospitalisation for acute medical illness (2 points), a history of VTE (1 point), and platelet count ≥350 × 10^9^/L (2 points) (Table 1) [22]. This score has been validated in ambulatory patients with lung cancer, but two different cut-off values for risk stratification have been used in different studies [23,24,60]. The percentage of high-risk patients was 90% and 71% with a cut-off value of 7 and 11, respectively. There was an obvious difference in the incidence of VTE between high- and low-risk groups, which was 10.8% vs. 6.6% (*p* value not reported) and 23.8% vs. 0% (OR 9.65 [95% CI 1.24–75.24], *p* = 0.031) in the models with the cut-off values of 7 and 11, respectively [23,60].

Compared to the Khorana score and its variations, the COMPASS-CAT score had a higher sensitivity, which was 83% and 100% with a cut-off value of 7 and 11, respectively. However, the specificity decreased to 35–51%, which means about one-half to two-thirds of patients who did not develop VTE had been classified as high risk [23,24]. The COMPASS-CAT score with the cut-off value of 11 showed a high discrimination with a C-index of 0.89 [23]; however, with the change in the cut-off value from 7 to 11 this model needs further validation.

The varying performance between these models may reflect the composite of the different categories of predictors and their weights. The COMPASS-CAT score only comprises one biomarker, that is platelet count [22], while the Khorana score, the PROTECHT score and the CONKO score also include leukocyte count and haemoglobin level; meanwhile, the COMPASS-CAT score also places more weight on cancer-related and patient-related risk factors for VTE than the Khorana score and its variations do [17,20,21].

### 3.4. Models Developed in Ambulatory Patients with Lung Cancer

A dynamic model developed in NSCLC patients by Alexander et al. involves only two biomarkers: baseline fibrinogen level and dynamic D-dimer levels at baseline and after one month [51]. This risk model is described as three conditions: baseline fibrinogen ≥ 4.0 g/L and baseline D-dimer ≥ 0.5 mg/L, baseline D-dimer ≥ 1.5 mg/L, and month-1 D-dimer ≥ 1.5 mg/L; each condition scores 1 point and a high risk is assigned if the patient is receiving chemotherapy and the score ≥1 [51]. Seventy-two percent of a cohort of outpatients with lung cancer had a high risk of thromboembolism, and the incidence of thromboembolism at 6 months was 26.5% vs. 0% in the high- and low-risk groups, respectively [51]. This model had a high sensitivity (100%, 95% CI 79–100%) but a low specificity (34%, 95% CI 23–47%), while the discriminating capacity was moderate (C-index 0.67, 95% CI 0.61–0.73) [50]. This model may not be applicable in patients with lung cancer who do not receive chemotherapy.

The ROADMAP-CAT model, which was developed in outpatients with lung adenocarcinoma, involves laboratory parameters only, which are procoagulant phospholipid-dependent clotting time (Procoag-PPL) and mean rate index (MRI) of thrombin generation [24]. This model is a binary scoring system: patients with Procoag-PPL < 44 s and MRI < 125 nM/min are identified with a high risk of VTE, while those with Procoag-PPL > 44 s or MRI > 125 nM/min are considered low risk [24]. The incidence of VTE in a 6-month follow-up period was 12.2% and 3.4% in high- and low-risk groups, respectively. The sensitivity was relatively high (88%), but the specificity was relatively low (52%), with a C-index of 0.77 showing moderate discriminatory performance [24].

## 4. Risk of Bias in VTE Risk Model Development and Validation Studies

We used the Prediction model Risk Of Bias ASessment Tool (PROBAST) [64] to identify potential biases in the development or validation of current VTE risk models. The first issue is the small sample size of many studies. As a rule of thumb, for risk model development studies, events per variable (EPV) should not be less than 10, while for risk model validation studies, there should be more than 100 participants with the occurrence of the outcome, in this case, VTE [65]. Secondly, dichotomisation of continuous predictors, such as white cell count, platelet count, BMI, D-dimer and fibrinogen, occurs in almost all models, which may lead to loss of linear information [65]. In addition, univariable analysis is a popular approach for predictor selection; however, this may miss some important predictors that are confounded by other predictors [65]. Furthermore, internal validation is necessary for directing an adjustment to build a robust risk prediction model, but in the VTE risk model development studies in ambulatory patients with lung cancer, internal validation was overlooked [65].

Model overfitting in risk model development could arise from issues such as small EPV, dichotomisation of continuous predictors, selecting predictors solely based on univariable analysis, or lack of internal validation with bootstrapping or cross-validation in the development studies. A lack of calibration is the next problem in both risk model development and validation. Calibration indicates the accuracy of a risk model by showing agreement between the expected number of events based on the risk model and the observed number of events [66]. Calibration is indispensable in the external validation of prognostic risk models, and calibration plots are even suggested at more than one time point for those models with competing risks [67]. Last but not least, there is use of a derived clinical score in risk models that does not reflect the actual weight of a predictor from the multivariable analysis in VTE risk model development [51]. The logistic regression equation is the actual expression of the risk model developed from the data [23].

Validation of VTE risk models should include patients receiving all currently approved medications, including anticoagulation therapies in this context. Ideally, a VTE risk model to be used in cancer patients should also provide some hint as to the most suitable medication to be used [68]. To address the growing clinical complexity, use of novel technologies, such as information-technology-powered decision support systems and Artificial Intelligence (AI) algorithms might be helpful both in the development and the validation phases. In this regard, preliminary studies have recently provided promising results [68,69].

## 5. Conclusions

Anticoagulant thromboprophylaxis in ambulatory patients with lung cancer could be a life-saving treatment by preventing VTE. However, since this treatment increases the risk of bleeding, it should be administered only in patients who are at a high risk of developing thromboembolic events. In this literature review, we have discussed some of the available risk prediction models for VTE which could be used in ambulatory patients with lung cancer. The original Khorana score and its modifications may not be sensitive enough to identify high-risk ambulatory patients with lung cancer. With the cut-off value changing from 3 to 2 points, the Khorana score may be effective, but it needs to be validated in ambulatory patients with lung cancer. The COMPASS-CAT score and some other developed risk models may be promising, but further validation is needed. Correct statistical methods and processes, including sufficient sample size, pre-specified predictor candidates, calibration plots and internal validation, should be considered and used in both risk model development and validation studies to ensure a low risk of bias.

## Figures and Tables

**Table 1:** Models for predicting VT E in ambulatory patients with lung cancer [17,20,21,22,23,24,25,51,56,57,58,59,60].

Name of Model (Author, Year)	Cancer Type for Model Derivation	Predictors	Score	High Risk	Validated By First Author, Year	Cancer Type for Model Validation
Khorana Score (Khorana, 2008) [17]	various	Cancer tissue:		Score ≥ 3 ^#^	Alexander 2019 [51]	NSCLC
		Very high-risk site (stomach, pancreas)	2			
		High-risk site (lung, lymphoma, gynaecologic, bladder, testicular)	1		van Es 2020 [58]	Various (lung cancer 58%)
		Platelet count ≥ 350 × 10^9^/L	1		Vathiotis 2018 [56]	Lung adenocarcinoma
		Haemoglobin < 100 g/L and/or use of ESA	1		Kuderer 2018 [25]	Lung cancer (84% NSCLC)
		Leukocyte count >11 × 10^9^/L	1		Rupa-Matysek 2018 [23]	Lung cancer (97/118 NSCLC)
		BMI ≥ 35 kg/m^2^	1		Mansfield 2016 [57]	Lung cancer (87.1% NSCLC)
PROTECHT (Verso 2012) [20]	various	As Khorana Score, but		Score ≥ 3	Alexander 2019 [51]	NSCLC
		**adds** gemcitabine chemotherapy, and	1		Rupa-Matysek 2018 [23]	Lung cancer (NSCLC 97/118)
		platinum chemotherapy	1			
CONKO (Pelzer 2013) [21]	various	As Khorana Score, but s		Score ≥ 3	Alexander 2019 [51]	NSCLC
		**removes** BMI ≥ 35 kg/m^2^, and			Rupa-Matysek 2018 [23]	Lung cancer (NSCLC 97/118)
		**adds** ECOG PS ≥ 2	1			
COMPASS-CAT (Gerotziafas 2017) [22]	Various (13% lung cancer)	Anthracycline treatment	6	Score ≥ 7	Spyropoulos 2020 [60]	Various (29.05% lung cancer)
		Time since cancer diagnosis ≤ 6 months	4			
		Central venous catheter	3			
		Advanced stage of cancer	2			
		Cardiovascular risk factors present	5		Syrigos 2018 [24]	Lung adenocarcinoma
		Hospitalisation for acute medical Illness	2	Score ≥ 11	Rupa-Matysek 2018 [23]	Lung cancer (97/118 NSCLC)
		A history of VTE	1			
		Platelet count ≥ 350 × 10^9^/L	2			
ROADMAP-CAT (Syrigos 2018) [24]	Lung adenocarcinoma	Procoag-PPL < 44 s, **and** MRI < 125 nM/min	1 ^†^	score = 1		
HS D-dimer (Ferroni 2012) [59]	Lung cancer	Khorana Score intermediate group **adds** high-sensitive D-Dimer		Khorana Score 1–2 and HS D-dimer ≥ 1500 ng/mL		
Model 1 (Alexander 2019) [51]	NSCLC	Baseline fibrinogen ≥ 4.0 g/L and baselie D-dimer ≥ 0.5 mg/L	1	Score ≥ 1	Underway ACTRN12618000811202 [51]	NSCLC
		Baseline D-dimer ≥ 1.5 mg/L	1			
		Month-1 D-dimer ≥ 1.5 mg/L	1			

^#^ Score ≥ 2 was used to stratify high-risk patients in the CASINI clinical trial of primary thromboprophylaxis and has been incorporated into ASCO Guideline. ^†^ binary scoring: score = 1 if Procoag-PPL < 44 s and MRI < 125 nM/min; score = 0 if Procoag-PPL > 44 s or MRI >125 nM/min. VTE: venous thromboembolism; ESA: erythropoiesis stimulating agents; BMI: body mass index; NSCLC: non-small cell lung cancer; ECOG PS: Eastern Cooperative Oncology Group performance status; Procoag-PPL: procoagulant phospholipid-dependent clotting time; MRI: mean rate index of thrombin generation; HS: high-sensitive.

## Data Availability

No data have been synthesised and reported in this study.
